# Insights into interfacial effect and local lithium-ion transport in polycrystalline cathodes of solid-state batteries

**DOI:** 10.1038/s41467-020-19528-9

**Published:** 2020-11-11

**Authors:** Shuaifeng Lou, Qianwen Liu, Fang Zhang, Qingsong Liu, Zhenjiang Yu, Tiansheng Mu, Yang Zhao, James Borovilas, Yijun Chen, Mingyuan Ge, Xianghui Xiao, Wah-Keat Lee, Geping Yin, Yuan Yang, Xueliang Sun, Jiajun Wang

**Affiliations:** 1 MIIT Key Laboratory of Critical Materials Technology for New Energy Conversion and Storage, School of Chemistry and Chemical Engineering, Harbin Institute of Technology, Harbin, 150001 China; 2 Department of Applied Physics and Applied Mathematics, Columbia University, NY New York, 10025 USA; 3 Department of Mechanical and Materials Engineering, University of Western Ontario, London, N6A 5B9 Canada; 4 National Synchrotron Light Source II, Brookhaven National Laboratory, Building 743 Ring Road, Upton, NY 11973 USA

**Keywords:** Batteries, Batteries, Batteries

## Abstract

Interfacial issues commonly exist in solid-state batteries, and the microstructural complexity combines with the chemical heterogeneity to govern the local interfacial chemistry. The conventional wisdom suggests that “point-to-point” ion diffusion at the interface determines the ion transport kinetics. Here, we show that solid-solid ion transport kinetics are not only impacted by the physical interfacial contact but are also closely associated with the interior local environments within polycrystalline particles. In spite of the initial discrete interfacial contact, solid-state batteries may still display homogeneous lithium-ion transportation owing to the chemical potential force to achieve an ionic-electronic equilibrium. Nevertheless, once the interior local environment within secondary particle is disrupted upon cycling, it triggers charge distribution from homogeneity to heterogeneity and leads to fast capacity fading. Our work highlights the importance of interior local environment within polycrystalline particles for electrochemical reactions in solid-state batteries and provides crucial insights into underlying mechanism in interfacial transport.

## Abstract (web summary)

Solid state battery is regarded as one of the most promising next generation energy storage systems due to high safety and high energy density. Here, authors demonstrate the importance of interfacial local environment in polycrystalline cathodes for electrochemical reactions in solid-state batteries.

## Introduction

Increasing demand for solid-state batteries has prompted intensive research into fast ion transport in solid-solid interfaces. Satisfactory solid-state battery performance requires the combination of highly ionic conducting electrolytes with low-resistance solid-solid interfaces^1^. State-of-the-art solid-state battery research no longer focuses on maximizing ionic conductivity in solid electrolytes, but has instead aimed to integrate the interfaces between electrodes and solid electrolytes^2^. This challenge in solid-state battery assembly is largely associated with maintaining chemical and mechanical stability between the electrodes and electrolytes during battery operation^3,4^. In traditional liquid electrolyte lithium-ion batteries (LELBs), the electrochemical reaction at solid–liquid interfaces involves soluble redox reactions, electroplating, and lithium intercalation processes, which has been well established based on the Butler-Volmer equations, Marcus theory, and adatom-reaction model^5^. The essentials of interfacial reactions at the solid-solid interfaces in all-solid-state lithium batteries (ASSLBs), however, are still ambiguous. There are few reports regarding the fundamental mechanisms that determine the solid-solid interfacial kinetics in the solid-state batteries.

The initially discontinuous or inhomogeneous physical contact is traditionally considered the principal hurdles for lithium transport across the solid-state interfaces. The inevitable physical contact loss may induce high impedance at the electrolyte–electrode interface that may degrade upon further cycling^5^. Unlike liquid electrolytes in conventional lithium-ion batteries that can easily diffuse across the interface and porous electrodes, solid electrolytes cannot access the voids and disconnections in solid-state batteries and therefore impedes synchronized lithium transport across the solid-solid interfaces^6^. Furthermore, the discontinuous physical contact and the resulting heterogeneous interfacial chemical property may evoke uneven  state of charge (SOC) and stress distribution, which significantly affect the interfacial stability during cycling. This may be worse for the polycrystalline cathode particles consisting of densely packed primary grains with rough surface^7^, in which the mesoscale architecture involves complicated ion diffusion through “outer” interfaces of electrolyte/particles and “inner” interfaces of grain boundaries, resulting in a high likelihood of physical and chemical evolution on the ion transport path^8^. Nevertheless, these insights into solid-solid reaction mechanisms are based largely on our conventional wisdom and general knowledge but lack of direct experimental evidence. Extensive efforts to fully understand the fundamental interfacial reaction mechanism in solid-state batteries are underway and the practical impact of physical contact on interfacial ion transport and interfacial electrochemistry remains to be proven.

In this work, we elucidate the fundamentals of physical contact loss in solid-state batteries and find that the initial physical contact of solid-solid interface shows a minor influence on solid-state battery performance. Contrary to our expectations, despite the remarkable physical contact loss, the initial capacity and Coulombic efficiency in a solid-state battery are found comparable to those of a conventional liquid-electrolyte lithium battery. Using synchrotron X-ray spectroscopic microscopy, we reveal that, in spite of inhomogeneous ionic transport at the solid–solid interfaces, cathode particles in solid-state battery still exhibit nearly full delithiation upon the finial charge state via the internal charge propagation driven by ionic-electronic fields, thereby delivering high electrochemical properties. The interior local environments in secondary particles therefore play a crucial role in determining ion transport for solid-state batteries. We further identify that the decay in solid-state batteries during cycling can be largely attributed to the microcracks within the polycrystalline particles, along with the severely deteriorated interfaces. Different from traditional work in the LELBs that cathode cracks were triggered by high operating voltage or fast charging/discharging rates, here a regular working voltage and discharge/charging rate was applied, so the generation of cracks may be attributed to the uneven interface reactions in the ASSLBs. We proposed that the persistent ion-transport kinetic nonequilibrium at the solid-solid interfaces accelerate the unbalanced electrochemical reactions, and therefore result in the stress heterogeneity and generation of microcracks. Our work highlights a previously overlooked behavior in solid-state ion transport, revealing the mechanism and effects of heterogeneous interfacial kinetics during the long-term cycling of all-solid-state lithium batteries.

## Results

### Electrochemical properties of solid-state batteries

**Fig. 1:**
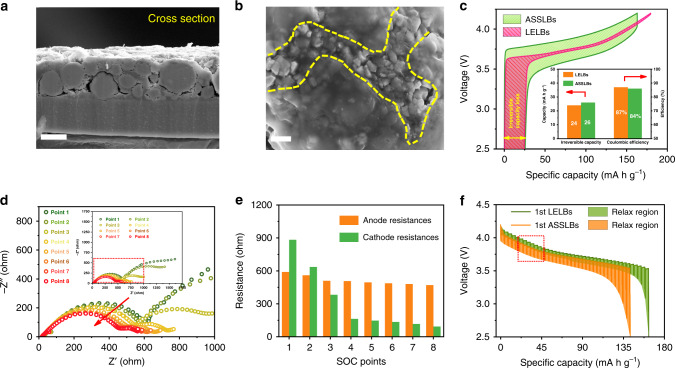
Electrode morphology, microstructure, and electrochemical behavior of NCM in the ASSLBs. (**a**, **b**) Typical SEM images of the cross-section and surface of the actual NCM electrodes in the ASSLBs. Scale bar, 10 μm, 2 μm. (**c**) Charging and discharging profiles of the NCM cathodes in the ASSLBs and LELBs at 0.2 C, where the inset shows irreversible capacities and initial Coulombic efficiencies of the ASSLBs and LELBs. (**d**) Evolution of the EIS results in the charging process, corresponding to the selected points in Supplementary Fig. 5a. (**e**) Fitting anode resistance and cathode resistance of the ASSLBs in the charging process. (**f**) GITT profiles of the NCM electrode in the ASSLBs and LELBs at different states of charge, respectively.

Commercial LiNi_0.6_Co_0.2_Mn_0.2_O_2_ (NCM) polycrystalline particles with an average size of approximately 10 μm were selected as a model material owing to its hierarchy configuration. Primary particles are randomly oriented, forming overall spherical secondary particles, as shown in Supplementary Fig. 1. Flexible solid polymer electrolyte (SPE) films based on polyethylene oxide (PEO) complexed with lithium bis(trifluoromethane sulfonimide) (LiTFSI) were prepared by solution casting method (Supplementary Fig. 2). Different from the full infiltration of liquid electrolyte in the electrode of LELBs, the discontinuous solid-solid contact between cathode particles and solid electrolytes often appears in the ASSLBs. As shown in Fig. 1a, b, most cathode particles are embedded in the solid-state electrolytes, but some edges of the active materials are not fully covered (Supplementary Fig. 3), which may affect the electrochemical potential at particle surface due to the absence of effective ionic paths. The heterogeneous electrochemical potential in the particles may evoke a heterogeneous interfacial reaction, triggering anisotropic ion transportation within the inner particles and affecting electrochemical kinetics. Therefore, we first utilized cyclic voltammetry (CV) to investigate the fundamental solid-state electrochemical reversibility. As shown in Supplementary Fig. 4, the peak potentials of oxidation and reduction in the ASSLBs present small shifts comparing to those in the LELBs. The wider half-peak width in the ASSLBs also verifies the presence of an uneven solid-state interface.

However, contrary to the expectations, we observed that the electrochemical performance of the solid-state battery is comparable to that of liquid-electrolyte battery. Figure 1c shows a comparison of the initial charging/discharging profiles at 0.2 C between ASSLBs and LELBs. Despite a slightly lower capacity and higher overpotential, the ASSLBs exhibit similar irreversible capacity loss (26 mA h g^−1^) to that of LELBs (24 mA h g^−1^). This can be further verified by similarity in Coulombic efficiency between ASSLBs (84%) and LELBs (87%) in the Fig. 1c inset, indicating the majority of lithium ions extracted in the initial charging process can return to the cathodes and therefore demonstrate the high reversibility in the ASSLBs. These unusual phenomena suggest that the initial battery performance in ASSLBs may not be inhibited by interfacial physical contact and the heterogeneous local environments at the interface regions. To evaluate the interface kinetics in the pristine ASSLBs, in-situ electrochemical impedance spectroscopy (EIS) was performed (Supplementary Fig. 5 and Fig. 1d). As shown in Fig. 1e, the EIS results present a decrease in total resistance during the initial charging of ASSLBs^9^. The two key parameters, anode resistance and cathode resistance, vary differently with the charging process. The anode interface resistances undergo a steady slow decline in the charging process. By contrast, the cathode interface resistances show significant decrease with the charging, which can be considered as a result of adequate activation of the pristine solid interfaces. The results support the establishment of stable solid-solid interfaces during the initial cycle, in spite of discontinuous physical contact at solid–solid interfaces.

Given that the integrated interface was formatted and activated in the initial charging process of ASSLBs, galvanostatic intermittent titration technique (GITT) was performed to measure macroscopic Li^+^ diffusion. In the GITT, a short negative current pulse was applied and then removed, followed by a sharp increase and gradual rise to achieve the thermodynamic equilibrium potential. Here, quick discharge voltage drops (ohm drop, IR) and slow decrease (ΔEτ) were shown in Supplementary Fig. 6a, representing an elimination of chemical composition inhomogeneity via Li^+^ diffusion droved by the electrochemical polarization and concentration polarization. Based on the formula $D_{Li^ + } = \frac{4}{{\pi \tau }}\left( {\frac{{m_BV_M}}{{M_BA}}} \right)^2\left( {\frac{{{\Delta}E_s}}{{{\Delta}E_\tau }}} \right)^2$, a series of $D_{Li^ + }$ versus SOC is generated. Note that the lithium metal anode produces a negligible overpotential, so the GITT transient response depends on the diffusivity of Li^+^ in the NCM cathode^10^. Due to that the relaxation overpotential of NCM cathodes is higher than that of LELBs (Fig. 1f and Supplementary Fig. 6b), the calculated lithium diffusion coefficient at each SOC point of the NCM materials is of the same order of magnitude as that of LELBs (10^−12^ cm^−2^ s^−1^ – 10^−11^ cm^−2^ s^−1^), as shown in the Supplementary Fig. 6c. Although fast lithium transport was restrained at high charging/discharging rates (Supplementary Fig. 6d), it can be deduced that the ion transport across the interfaces in ASSLBs may be slightly influenced at low charging/discharging rates, which is ascribed to the adequate relaxation time to achieve a concentration equilibrium under the driving force from electric fields and concentration difference.

### Effects of solid–solid interface on the local lithium-ion transport

It is traditionally accepted that lithium-ion transportation through the solid–state interfaces is susceptible to the discontinuous interface contact, high diffusion barrier, and chemically unstable interfacial status^11^. The above electrochemical measurements, however, suggest that the interfacial contact loss may only show limited influence on the solid-state battery electrochemistry. It is clear that the polycrystalline particles consist of numerous constituent primary grains and grain boundaries, and therefore the redox reactions usually do not occur concurrently in single grain. As a result, the ionic transport in secondary NCM particles may influence the electrochemical reaction in the grain of cathode particles differently for solid-state batteries.

In order to determine the underlying ion transport behaviors, we employ in-operando transmission X-ray microscopy (TXM) to probe the possible correlation of local interface environment with initial electrochemical property in solid-state batteries. The hard X-rays enable imaging of micrometer-thick materials, while the tunable incident photon energy provides information on the chemical states through spatially resolved XAS with a resolution of ∼30 nm (Supplementary Fig. 7a)^12^. The chemical mapping was quantitatively analyzed based on the TXM-XANES fitting, where the reference spectra (LiNi_0.6_Mn_0.2_Co_0.2_O_2_ and Ni_0.6_Mn_0.2_Co_0.2_O_2_) were obtained from the pristine sample and fully charged sample. As shown in Supplementary Fig. 7b, c, a clear shift of 3 eV occurs at the absorption edge of nickel when charging the cell from open-circuit state to 4.2 V. After acquiring the XANES data at each state of charge, chemical information at each pixel can be obtained by fitting the results with the two reference spectra^13^. Here, it is noted that nickel element undergoes a significant energy shift at absorption K-edge^14^. Therefore, in this work we selected the K-edge absorption edge change of Ni as an indicator for state-of-charge of battery materials.

**Fig. 2:**
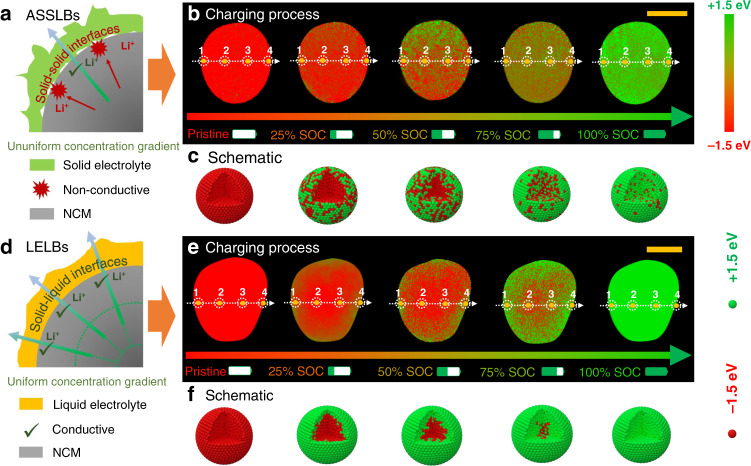
In operando 2D TXM-XANES mapping during the initial charging process. (**a**) Schematic of solid-solid interface models and kinetics. (**b**) TXM-XANES mapping of single cathode particle as a function of charging time in ASSLBs, and (**c**) the corresponding schematic diagram to expound the unique solid-state electrochemistry. (**d**) Schematic of solid-liquid interface models and kinetics. (**e**) TXM-XANES mapping of single cathode particle as a function of charging time in LELBs, and (**f**) the corresponding schematic diagram expound the conventional solid-liquid electrochemistry. Scale bar, 10 μm.

Figure 2a, b show the schematic of particle/electrolyte solid interface kinetics and chemical state evolution in 2D projected local state of charge of polycrystalline particle by performing spectromicroscopy at the Ni K-absorption edge as a function of charging time. In the early stage of charging, the changes of the chemical state of nickel occur at the partial particle surface, suggesting the lithium deintercalation starts from the surface and increasingly moves into the particle bulk. With continued charging, the chemical driving force makes the Li^+^ vacancy gradient propagate gradually towards the inner bulk. Note that the gravel-like packing of the grains creates random and likely tortuous Li-ion pathways, therefore the moving chemical state seems to be relatively anisotropic. Here, we notice that the discontinuous solid-solid contact or voids at the interfaces do not seem to interrupt the integrated interfacial ion transportation. Despite anisotropic ion transportation in the bulk phase and selective lithium ion transport through the interfaces, the chemical state of the particle presents relatively uniform distribution across the entire particle in the end. The explanation for this is that the unsynchronized lithium-ion concentration fields in the bulk phase is released by the combination of electrochemical force from electric fields and spontaneous diffusion under concentration gradient. An effective equilibrium of electron and local ionic concentration may make up the deficiency within initial physical contact loss at solid-solid interfaces, as schematically shown in Fig. 2c.

In detail, all electronic and ionic transport processes are caused by the chemical composition gradients and diffusion pathways in the local environment of battery materials^15^. Based on the solid-state transport principle, the kinetics of ionic/electronic transport is proportional to the sum of ionic and electronic resistance in battery materials. If either of ionic or electronic conductivity is low, the diffusion process in the electrochemical reaction slows significantly. The layer oxide NCM in this study is a mixed conductor with a high ionic and electronic conductivity^16^, therefore the bulk diffusion in NCM particles is not limited. The surrounded solid-state electrolyte, however, shows a lower ionic conductivity (10^−4^ S cm^−1^) than that of NCM bulk material (10^−3^ S cm^−1^)^17^. Therefore, the total lithium transport rate is largely determined by the low diffusion coefficient of solid-state electrolyte and the distance over which lithium-ion transport takes place. Initially, lithium extraction across the solid–solid interfaces is significantly inhibited by the high interfacial energy barrier, restricted solid–solid contact and low ion conductivity of solid-state electrolyte, leading to an unequilibrium of local ionic concentration gradient and electric fields^18,19^, which, in turn, can promote ion transport within the particles in the form of high overpotential. As the delithiation proceeds deeply, enough driving forces gradually move boundary of state of charge with obvious anisotropy, which can still nearly fully delithiate cathode particles, although the NCM particles were partially covered by solid electrolytes. As shown in Supplementary Fig. 8, the particle presents a roughly homogeneous chemical mapping after charging 60 min, 90 min, and 120 min, illustrating the fully charged polycrystalline particles in solid-state batteries.

In contrast to solid-state batteries, the conformal solid-liquid interfaces surrounding the surface of NCM particles can ensure a homogeneous and unrestricted interfacial ion transportation in the LELBs, enabling no stagnation of lithium ions to pass across the solid–liquid interfaces by taking advantages of high ionic conductivity and wettability of the interfaces (Fig. 2d). Driven by that, a nearly whole annular lithium-poor region on the particle surface can be formed initially. In addition, the kinetically rapid ion transport at the solid–liquid interface can significantly decrease the unequilibrium of the local ion-electron environment, which is beneficial for synchronized electron/ion equilibrium in the NCM bulk^20^. With the charging proceeds, lithium vacancies can diffuse toward the inner bulk and gradually spread into the center of a particle along the radial direction under the homogeneous annular concentration gradient, displaying an explicit “core–shell” delithiation model (Fig. 2e, f)^21,22^. The information in Supplementary Fig. 9 quantifies the chemical mapping of the particles with different SOCs. From the above comparative studies for LELBs and ASSLBs, despite the different ionic transport model of “core-shell” and homogeneity, both samples show homogeneous charge distribution within the finally charged particles. It is notable that the discontinuous interfacial ion pathway in ASSLBs results in incomplete lithium accommodation in polycrystalline cathode particles at the beginning and intermediately charged state, as expounded in the schematic of ASSLBs. However, the unbalanced local ion-electron effects can induce a strong driving force to nearly completely delithiate the cathode particles, therefore delivering relatively comparable capacity and reversibility as that of the LELBs. It is generally known that lithium-ion diffusion is always associated with the electron transfer process in lithium batteries^23^. The internal electric field distribution can drastically enhance lithium ions migration kinetics. Once the ion-electron equilibrium is changed, the electrochemical reaction dynamics can be reduced via potential polarization, concentration polarization, and side reactions, etc^24^. In this study, the dynamic chemical imaging upon the initial charging process reveals the existence of local ion concentration equilibrium in cathode of solid-state battery, in spite of substantially incomplete solid-solid contact between the particles and electrolyte at short length scales. It is therefore crucial to maintain stable local environment equilibrium for persistent solid-state battery properties.

### Solid–solid interface evolution upon cycling

**Fig. 3:**
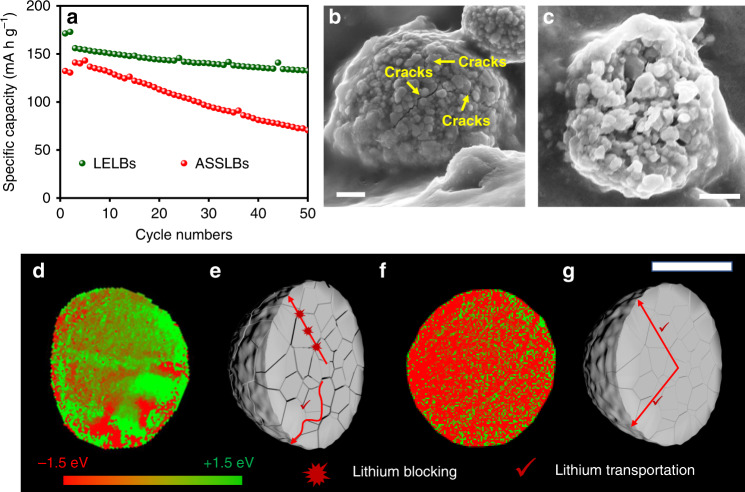
Structure and SOC distribution of the cycled cathode particles. (**a**) Comparison of the cycling stability of NCM electrodes in the ASSLBs and LELBs at 0.5 C after activation. (**b, c**) Typical SEM images of the cycled NCM electrodes in the ASSLBs, scale bar, 2 μm, 3μm. (**d**) 2D TXM-XANES mapping of cycled NCM particle in the ASSLBs, and (**e**) diagram of the effects of cracks on lithium transportation. (**f**) 2D TXM-XANES mapping of cycled NCM particle in the LELBs, and (**g**) diagram of the slight effects of microcracks on lithium transportation. Scale bar, 10 μm.

Although the initially homogeneous lithium-ion transport dynamics can be relieved by appropriate ionic transport balance in the inner particles, it is hardly sustained upon cycling. As shown in Fig. 3a, after 50 cycles, the LELBs can maintain high capacity retention of 85%, while the ASSLBs exhibit significant performance degradation with a capacity retention of 51%. Further, the increased voltage polarization and battery resistance are responsible for the different performance degradation (Supplementary Fig. 10, Supplementary Fig. 11). Here, observation of the morphology of cycled cathodes verifies the presence of voids at the solid–solid interfaces (Supplementary Fig. 12) and microcracks in NCM particles (Fig. 3b, c). We, therefore, suspect that the performance loss may be associated with the deteriorated interfacial properties in solid-state batteries, which generally involve the degenerated electrode/electrolyte interfaces and the broken cathode particles in the solid-state battery^25^.

**Fig. 4:**
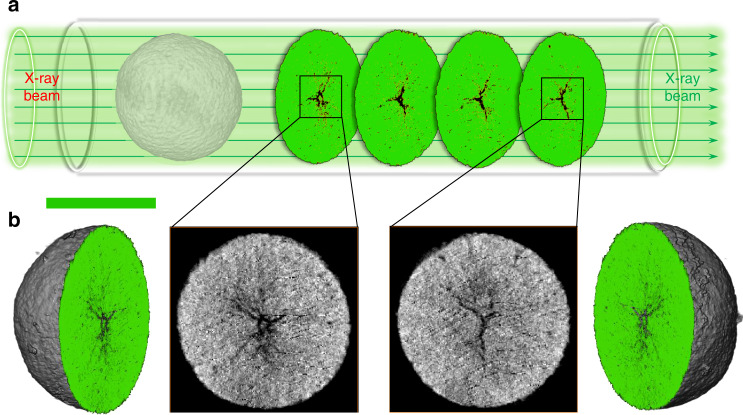
X-ray nanotomography of the cycled cathode particles in the ASSLBs. (**a**) 3D renderings and virtual slices of the polycrystalline NCM particle structure after 50 cycles. (**b**) Representative slices through different depths of the particle. Scale bar, 10μm.

To develop insight into how interfacial issues affect cathode particles during cycling, we studied chemical charge distribution within solid-state battery after 50 cycles. In contrast to the homogeneous charge distribution in the initial charge process, the cycled cathode particles display heterogeneity and significant SOC separation in ASSLBs (Fig. 3d). By comparison, the cycled cathode particles in the LELBs (Fig. 3f) maintain their chemical homogeneities. We, therefore, consider the heterogeneous charge map in solid-state battery materials as the gradual failure of ion transportation paths caused by the continuous physical contact loss and the irreversibility of lithium transport across the interfaces. It is well-known that deintercalation of Li^+^ in a layered transition metal oxide usually causes lattice expansion along the *c* direction and contraction along with the *a* and *b* directions^26^. In particular, for polycrystalline NCM particles with micron size, a number of grain boundaries and pores throughout the entire particle can further exacerbate microstructural change. Consequently, the persistent volume expansion/contraction and mechanical deflation may result in increased interfacial ion transport resistance, inducing the charge heterogeneity of cathode particles in the solid-state battery. Furthermore, the anisotropic lithium concentration changes and phase separation may evoke accumulated inherent stress and finally form a number of microcracks within the polycrystalline particles (Fig. 3e)^14^. Relatively, the well electrolyte penetration in the LELBs is a favor to the lithium migration and homogeneous stress distribution across the particle, resulting in slight stress and damage along the grain boundaries (Fig. 3g). As shown in Fig. 4a, b, the 3D rendering of the NCM particles display the different views of slices in the solid-state battery materials, where the obvious microcracks are observed in the particle, which is consistent with the SEM images (Supplementary Fig. 13). The fresh exposed surfaces or interfaces induced by microcracks are inaccessible to the solid electrolytes with nonfluid characteristics, aggravating the phase heterogeneity, interfacial contact loss, and even the final pulverization of polycrystalline particles. In contrast, no obvious microcracks were observed in the SEM images of cycled electrodes in the LELBs, as shown in Supplementary Fig. 14. The nanotomography of the particle was also performed in the electrodes of the LELBs after 50 cycles (Supplementary Fig. 15), where cracks were absent from the 3D rendering of the particles along with the slices. Therefore, polycrystalline particles in the LELBs can keep the intrinsic microstructure intact after 50 cycles.

**Fig. 5:**
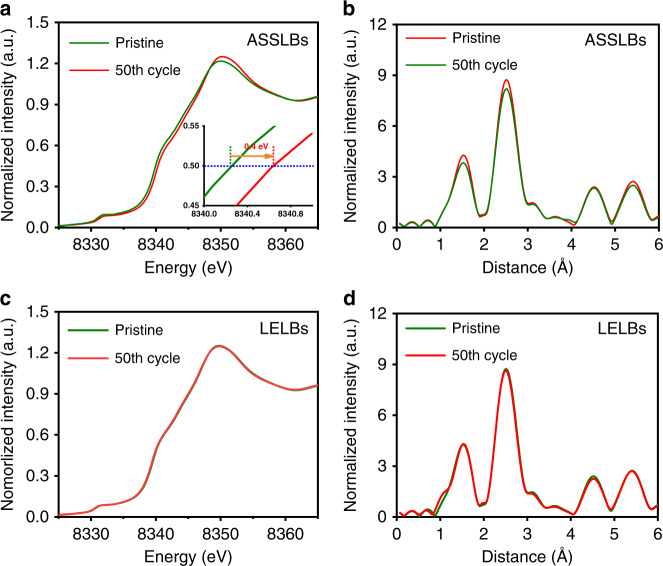
Evolution of chemistry information of cycled NCM cathodes in the ASSLBs and LELBs. XANES and EXAFS of the pristine electrodes and cycled electrodes after 50 cycling (**a**, **b**) in the ASSLBs, and (**c**, **d**) in the LELBs.

In addition to SOC heterogeneity, the increased interfacial resistance and incrementally sluggish interfacial kinetics also create highly structural irreversibility in cathode particles of solid-state battery. In order to establish the relationship between the solid–solid interfaces and phase transformation of NCM particles, we performed synchrotron X-ray structural analysis for cycled battery materials. XRD analysis of the cycled electrodes in solid-state batteries showed a significant negative shift of 0.04^o^ compared with the pristine samples, indicating an increase in interplanar layer distance (Supplementary Fig. 16a). The chemical status was further investigated using the K-edge XANES (Fig. 5a, c). In contrast to the negligible valence state change of nickel in LELBs, the cycled electrode in the ASSLBs presents a positive shift of 0.4 eV at K-edge, indicating a remarkable increase in nickel valence in cycled cathode particles. More detailed information is shown in Supplementary Fig. 16b. As for the cycled particles in the ASSLBs, the XANES absorption edge of the core region presents a positive shift compared with that of interfacial region (Supplementary Fig. 16c), indicating a heterogeneous SOC distribution in spatial dimensions which is triggered by the disconnected ion channel arising from cracks after cycles. By comparison, the core region and the interface region display similar X-ray absorption spectra in LELBs (Supplementary Fig. 16d), representing the homogeneous SOC distribution all over the particles. Furthermore, from the extended X-ray absorption fine structure (EXAFS), the amplitude for the Ni-O and Ni-M peaks of NCM in the LELBs changed slightly (Fig. 5b). Different from that, the amplitude for Ni-O and Ni-M peaks in the ASSLBs was significantly reduced after cycling (Fig. 5d), revealing that the octahedral coordination of the pristine NiO_6_ environment was distorted by the excess Ni^3+^ (a Jahn–Teller active (d7) ion). A decrease in the cation ordering of LiNi_6_ in transition metal layers was also verified by the dramatic drop of Ni–M peak^27^.

## Discussion

**Fig. 6:**
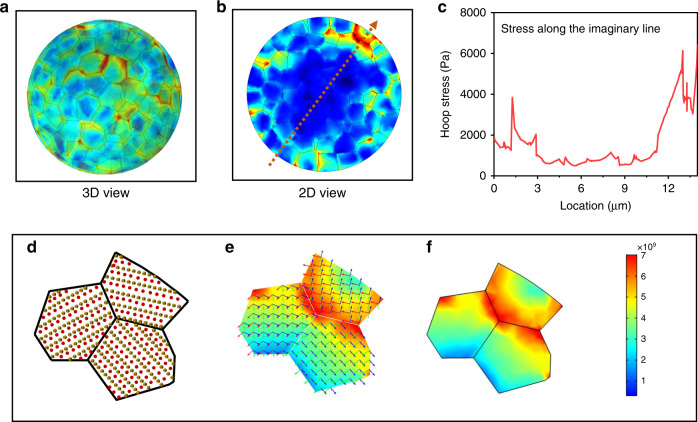
Finite element modeling of the cathode polycrystalline particles. The equivalent stress within the NCM particles upon completion of charging (Li extraction) of ASSLBs. (**a**) three-dimension view with 50% transparency (**b**) two-dimensions view of cross-section, and (**c**) stress distribution within the NCM particles along the imaginary line in Fig. 6b. Diagram of heterogeneous mechanical stress distribution in the select district. (**d**) different crystalline orientation, (**e**) different lithium diffusion orientation, and (**f**) stress distribution in the primary particles.

To understand the influence of interfacial behaviors on cathode particle properties in the ASSLBs, we employed finite element modeling to investigate the interfacial chemo-mechanics-induced stress evolution within NCM polycrystalline particles. Theoretical models of NCM polycrystalline particles in the ASSLBs were built using aggregated polygonal primary grains with random orientation, where the surface regions of particles were constructed with partial non-reactive grains considering the practical operating condition. Figure 6a shows the half-transparent view of stress distribution within the partially charged particles (50% SOC) in the ASSLBs, where heterogeneous stress distribution was exhibited in both tension (red) and compression (blue). Stress along the radial direction of the NCM particles was shown as a function of particle radius in Fig. 6b, c. The origination and mechanism of local stress along the grain boundaries are detailly shown in Fig. 6d–f, where the anisotropic crystal orientation and lithium diffusion are mainly responsible for the results. During delithiation, lower Li concentration in the outer shell than the core region induces a Li concentration gradient, causing the polycrystalline particle to experience tensile stress near the surface and compressive stress at the center. Here, the unbalanced concentration distribution between the surface region with depleted Li^+^ species and the core region with high Li^+^ contents lead to the uneven spatial distribution of stress at the NCM particles. It is probable that the nonequilibrium in repeated charging/discharging will destroy particle structure and accelerate battery performance degradation^28^. By contrast, the LELBs deliver a low variation of stress distribution within the particles (Supplementary Fig. 17), indicating that the mechanical disintegration takes places in the NCM particle for ASSLBs^29^. With the persistent charging to 100% SOC, the more intense variation of stress distribution all over the particles are presented in Supplementary Fig. 18. We may conclude that the non-uniform stress field is derived from unequilibrated charge distribution and therefore causes sluggish ion-diffusion kinetics in the polycrystalline particles of ASSLBs, facilitating the initiation, propagation of intergranular microcracks and irreversible degradation of battery performance.

Since the stress distribution within cathode particles is directly linked to lithium transport, we emphasize the critical role of the interior localized environment within cathode particles for lithium-ion transport in solid-state batteries. The discovery of minor electrochemical differences but distinct physical contact properties between ASSLBs and LELBs motivates us to uncover the underlying reaction mechanism in the particles. Owing to the rigid nature of solid electrolyte and mechanical artifacts during solid-state battery assembling, solid-state batteries are often accompanied with partial physical contact loss^30,31^. Nevertheless, regardless of intimate interfacial contact or partial physical contact, it may not necessarily determine solid–solid interfacial electrochemistry at the initial cycle. In contrast to the liquid electrolyte that is easily accessible to all the primary grains within polycrystalline particles via liquid penetration, solid electrolytes can only contact a small number of primary particles at the surface of secondary particles. As a result of closer contact with solid electrolytes, some regions within a particle may delithiate faster than their surrounding areas. Ultimately, most primary particles can achieve equilibration of the chemical potentials via internal lithium-ion propagation driven by the concentration gradient and potential polarization, regardless of whether the particle is fully covered by solid electrolyte or not^32^. Long cycles may lead still to unrecoverable interface failure, deteriorating solid-state battery performance. Full interface separation from solid electrolytes or irreversible interfacial reactions will decrease the utilization of active particles and primary grains. Although it was known that the oxidation of PEO is one of the most reasons responding for the battery failure, here we would like to emphasis that large cracks and severe pulverization in polycrystalline particles upon cycling also significantly increase the local ionic transport barrier within grain boundaries, further leading to an increasing resistance and fast degradation of ASSLB capacity.

From a mechanical point of view, the severe interfacial issues and ionic transport challenges can be resolved via materials and electrode design. Battery active materials can be tailored toward the desired physical/chemical properties for the desired local ionic concentration equilibrium in solid-state batteries. Single crystalline particles or polycrystalline particles with a dense structure to eliminate grain boundary resistance are critically important for solid-state battery reaction^33^. The mechanical properties such as plasticity and flexibility must also be considered as crucial criteria for solid-state electrolyte in future studies, which compensate for volume changes and mechanical loss in solid-state batteries upon cycling. Furthermore, the selection of appropriate electrode components and fabrication of an electrode with 3D ionic-electronic transport also plays a crucial role in solid-state batteries. An effective local ionic transport network to maximize solid-state electrochemical reaction and minimize irreversible side reactions merit significant work, as recent studies indicate that conductive carbon may also accelerate the decomposition of solid-state electrolyte^34^. Considering that severe mechanical effect and interfacial contact loss are largely attributable to electrode volume change, an ongoing search for zero-strain electrode materials to inhibit volume change-induced issues is also necessary for solid-state batteries.

In summary, we have elaborated on the unique solid-state lithium batteries electrochemistry from the insights into solid interfaces and local ion diffusion in the polycrystalline particles. In contrast to the traditional assumption of inferior electrochemical performance of solid-state batteries due to partial physical contact loss, NCM exhibit comparable electrochemical reversibility in a solid-state battery to that of a liquid-electrolyte battery, suggesting discontinuous physical contact leads to a slight difference in initial electrochemical behavior of polycrystalline particles compared to that in conventional liquid-electrolyte lithium batteries. Further, the local Li^+^ transport pathway was unveiled at scales varying from micrometers to single particles. The study revealed an unexpected homogeneity of the local Li concentration in a particle during initial charging, which added our insights into the existing knowledge of nonequilibrium electrochemical reactions in solid-state electrochemistry. The case could not be sustained, however, due to the continuously deteriorating interfacial issues upon cycling. Cathode particles in solid-state battery exhibit obviously heterogeneous SOC distribution and rapid capacity fade. 3D nano-tomography images and model calculations based on the discrete reaction interfaces verify the existence of cracks triggered by the uneven stress distribution along the primary grain boundary, accelerating the SOC heterogeneity in the cycled particles. Our investigation visualizes the close correlation between the interior local environments of cathode particles with solid-state interfacial behaviors and is expected to pave the way for engineering design and optimization of solid-state lithium batteries for future-generation electrochemical energy storage.

## Methods

### Materials and battery assembly

For an in-operando experiment, a special model cell was designed for synchrotron monochromatic X-rays transmission^35^. Commercial LiNi_0.6_Mn_0.6_Co_0.2_O_2_ powder (Zhuhai Coslight Corp.) was mixed with acetylene black and polyethylene oxide/LiTFSI at the weight ratio of 50:30:20 in acetonitrile solvent to prepare the slurry. The resulting slurry was pasted on commercial carbon paper and dried at 60 °C under vacuum for 72 h as a working electrode. Note that the slurry thickness was adjusted in a very thin scale to form a single-particle layer, which can ensure good electronic and ionic contact for the electrochemical reactions in the in-operando experiments. Then, the ASSLBs (coin cells, CR2025) with a hole window were assembled in argon glove box^36,37^, with lithium metal as anode and PEO (molecular weight, 5,000,000 g mol^−1^) as the solid electrolyte without the addition of any liquid-based electrolyte. As shown in Supplementary Fig. 19a, synchrotron monochromatic X-rays can directly transmit through a perforated 2025-type coin-cell containing the NCM cathode and all the other key components of a realistic battery, such as lithium foil, metal filler, and solid PEO electrolytes (Supplementary Fig. 19b). Here the measured NCM particles are in the central point of the electrode with highly reactive activity. Also, the holes in the coin-cell are sealed using Kapton tape to be isolated in the air atmosphere. The assembly procedure of LELBs is the same as that of ASSLBs, except that the solid electrolyte was replaced by liquid electrolyte consists of LiPF_6_ (1 mol L^−1^) dissolving in the solvent of EC/DEC/DMC (1/1/1, vol.%).

The coin cells for electrochemical measurements were assembled with a distinct approach as follows. The same LiNi_0.6_Mn_0.6_Co_0.2_O_2_ powder was mixed with acetylene black and polyethylene oxide with weight ratio of 80:10:10 in acetonitrile solvent. The resulting slurry was pasted on Al foil and dried at 60 °C under vacuum for 72 h as working electrode. Then, the ASSLBs (coin cells, CR2025) were assembled in argon glove box where lithium metal as anode and PEO as the solid electrolyte without addition of any liquid-based electrolyte. The assembly procedure of LELBs is the same as that of ASSLBs, except that the solid electrolyte was replaced by liquid electrolyte consists of LiPF_6_ (1 mol L^−1^) dissolving in the solvent of EC/DEC/DMC (1/1/1, vol.%).

### Electrochemical measurements

Galvanostatic charge/discharge measurement of the Li|PEO+LiTFSI|NCM cell was performed on the Neware battery test system (CT-4000) in the potential range of 3.0 V - 4.2 V at 60 °C. Cyclic voltammetry was performed in the electrochemical workstation (CHI660E instrument) with the same potential windows at 0.2 mV s^−1^ to investigate the battery chemistry. EIS measurements were also carried out in the CHI660E instrument and in the frequency range from 0.01 to 100,000 Hz. It should be noted that the in-situ EIS measurement is performed at the charging process of Li|PEO+ LiTFSI |NCM cell at 0.2 C at 60 °C, and the data were collected at equal time interval of 30 minutes. The working temperature of LELBs is room temperature 25 °C. GITT measurements were conducted in the versatile multichannel potentiostat system during the discharging process. The cells were set to relax for 2 h after every 30 min at discharging/charging rates of 0.1 C.

### In-operando TXM study

The TXM imaging with hard X-ray was conducted at beamline FXI-18, NSLS-II, BNL. A nanotomography with TXM is capable of analyzing the 3D morphology of the area of interest within the cell through the reconstruction of particles in the test electrochemical cell. The nanotomography data set was collected with 8 keV X-rays, using 361 projections over an angular range of 180°, along with a field of view of about 40×40 μm^2^ with a 2 k×2 k CCD camera binning 2×2 camera pixels into one output pixel. The resolution of this TXM has been quantified a sub-50 nm 3D resolution. Before reconstruction, the raw data was corrected using a run-out correction system building in the sample stages for automatic tomography alignment available at beamline FXI-18, NSLS-II^38^.

The in-operando 2D TXM-XANES imaging was performed on the above-mentioned model coin cell during the initial charging (delithiation) process. To study the chemical state evolution, a full XANES image series was collected at each charging stage during the delithiation process. Each XANES image series was measured by scanning Ni absorption K-edge from 8,033 to 8,055 eV, with 2 eV step size, and one TXM image at one energy step. Each image was collected with 20 ms exposure time. Camera pixels (2×2) were binned into one output image pixel. The 2D TXM-XANES data was fitting by the spectrum from standard samples to obtain the final SOC mapping^39^.

### Computational method details

The finite element modeling (FEM) was employed to study the stress distribution and mechanical failure of NCM particles during cycling. The models were built through aggregating polygonal primary particles with a random generation of shape, size, and orientations using the Voronoi tessellation. Here, considering that the electrochemical reaction occurs on the surface of the NCM particles, the source/sink term to describe electrochemistry is used as a boundary condition in the governing equation for the Li diffusion to calculate the flux size $\left( {J\left( {r_p,t} \right) = \frac{{j_{loc}}}{F}} \right)$ on the surface of the active particles, where the *j*_*loc*_ represents the local current density and the local current density is assumed to be a constant value. Further, the mathematical coupling of mass transfer and mechanics is in a stepwise process. First, the distribution of lithium-ion concentration *c* in the active particles can be calculated after the surface flux is applied. Then, given that the stress-strain response is faster than the mass transfer process and can be regarded as a quasi-steady-state process, the initial value calculated in the first step can be directly used to calculate the stress/strain distribution across the particles. In the ASSLBs, the surface of NCM particles is subject to a partial Li outflux through randomly setting 66.7% particles reactive but 33.3% particles unreactive. In the LELBs, the surface of NCM particles is subject to a complete Li outflux through setting 100% particles reactive. All the magnitude is calculated from a galvanostatic charging rate of 1 C. It should be noted that the Li diffusion kinetics in cathode particles follows the Fick’s law $\frac{{\partial {\mathrm{c}}}}{{\partial t}} = D\nabla ^2c$, where *c* and *D* are the Li concentration and Li diffusivity in NCM, respectively. In the simulation of stress distribution, the anisotropic deformation of NCM has been considered, where the diffusion coefficient of lithium along the [100], [010], and [001] directions was 5 × 10^−15^, 5 × 10^−15^ and 1 × 10^−20^ m^2^ s^−1^.

The detailed calculation process is as follows. The total strain here is the sum of two components, chemical ($\varepsilon _{ij}^c$) and elastic ($\varepsilon _{ij}^e$) one, as $\varepsilon _{ij} = \varepsilon _{ij}^c + \varepsilon _{ij}^e$, where the subscripts *i*, *j* = 1, 2, 3 represent the [100], [010], and [001] directions of particles. The chemical strain is evoked by lithium extraction, which is assumed to be proportional to the normalized lithium concentration (c) by a fully lithiated state, as $\varepsilon _{ij}^c = \beta _{ij}c$. The diagonal tensor, *β*_*ij*_, represents the lithiation expansion coefficients. As for NCM, we set *β*_11_ = *β*_22_ = 2.8%, *β*_33_ = −4.0%, and *β*_*ij*_ = 0 for the other entries^40^. As an orthotropic crystal, the stiffness tensor of the layered structure depends on nine independent material constants. We set the material constants of NCM (*c* = 0) and LiNCM (*c* = 1) in the model, as shown in Supplementary Table 1^41–43^, and assume that the stiffness tensor of the intermediate stages linearly scales with the lithium concentration (*c*).

Finite element software COMSOL 5.3 is used to solve the co-evolution of Li concentration and mechanical stresses in NCM particles^44^. Meshing procedure for the simulations is operated as the following parameters. The free tetrahedral grid is applied to build the mesh, which is composed of 282002 tetrahedral elements, 49281 triangular elements, 8149 edge elements, and 1817 vertex elements. For this study, the minimum mesh quality is 0.196, and the average mesh quality is 0.662.

## Supplementary information

Supplementary Information

## Data Availability

The data supporting the findings of this study are available within the article and its Supplementary Information files, or from the corresponding authors on reasonable request.
