# Supplementary material for: Insights into interfacial effect and local lithium-ion transport in polycrystalline cathodes of solid-state batteries
Source: Nat Commun. 2020 Nov 11;11:5700. doi: 10.1038/s41467-020-19528-9 (PMC7658997; doi:10.1038/s41467-020-19528-9)
Supplement: Supplementary file 1 — Supplementary Information [file 41467_2020_19528_MOESM1_ESM.pdf]

Supplementary Information

Insights into interfacial effect and local lithium-ion transport in polycrystalline cathode of solid-state batteries

Lou et al.

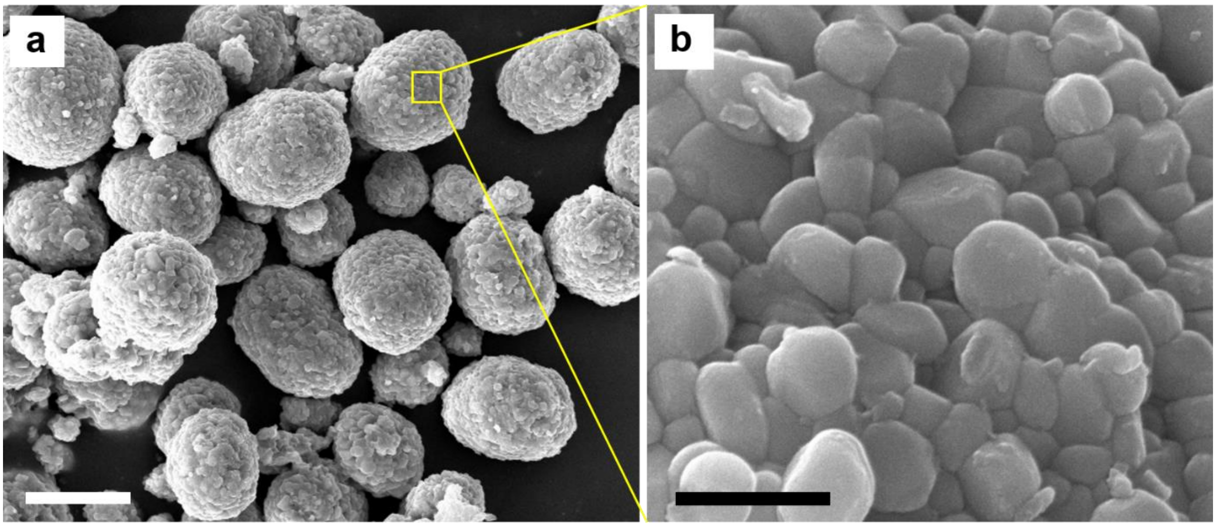

Supplementary Figure 1. Morphology of the pristine NCM particles. SEM images of NCM particles with different magnifications. (a) Low magnification. Scale bar, 10 μm. (b) High magnification. Scale bar, 1 μm.

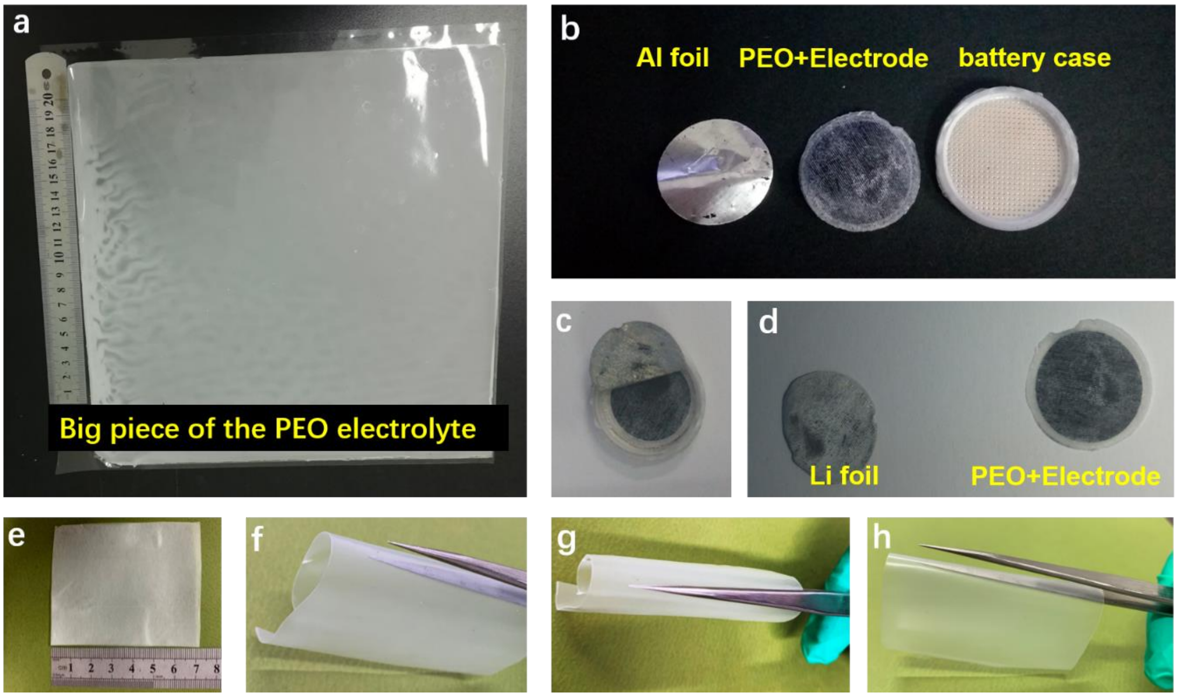

Supplementary Figure 2. Digital photograph of prepared PEO electrolyte and experimental batteries. (a) As-prepared big piece of PEO electrolyte. (b-d) Disassembling experimental battery. (e-h) high flexibility of the prepared PEO electrolyte.

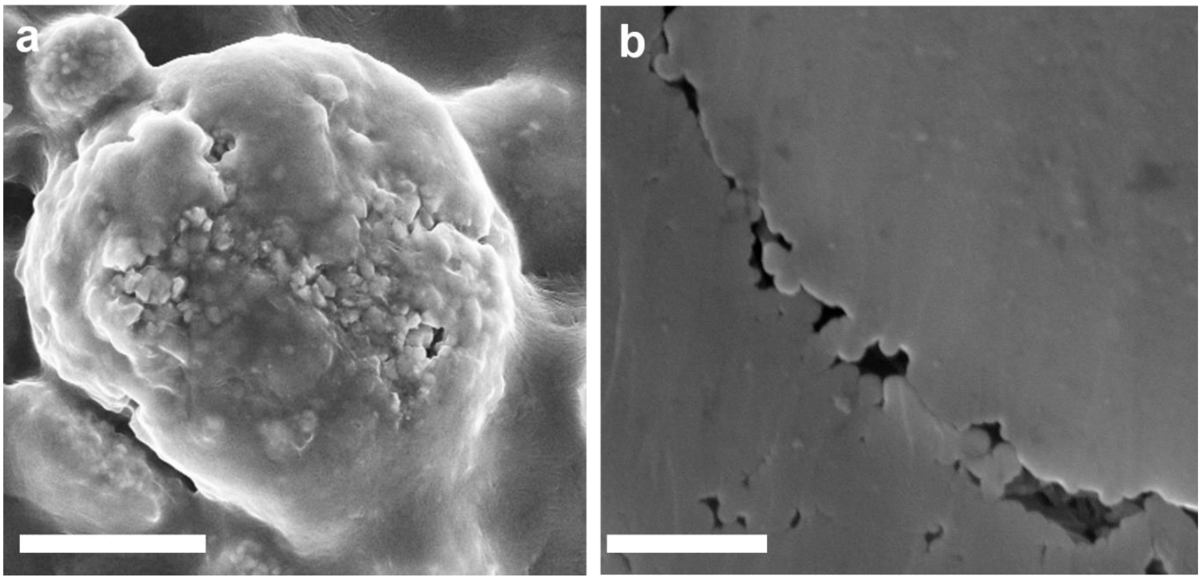

Supplementary Figure 3. Discontinuous contact of solid-solid interface between NCM particles and PEO SSEs in the pristine electrodes. (a) A representative particle with uncovered interface coverage of SSEs. Scale bar, 10 µm. (b) Cross-section SEM image of the contact loss state. Scale bar, 1 µm.

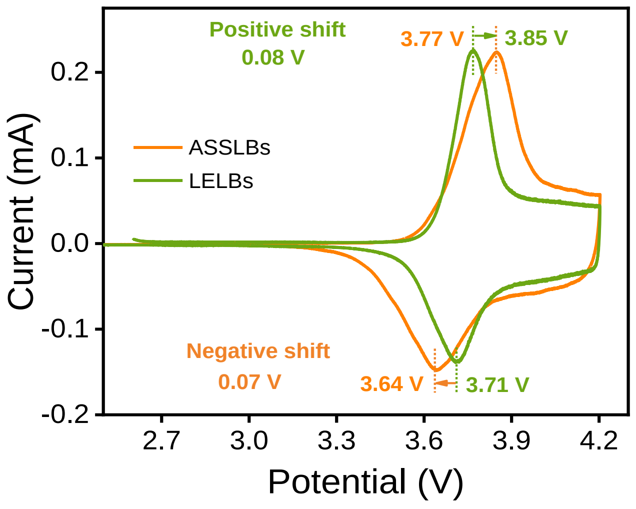

Supplementary Figure 4. The initial CV curves of the ASSLBs and LELBs at scanning rate of 0.2 mV s^{-1}.

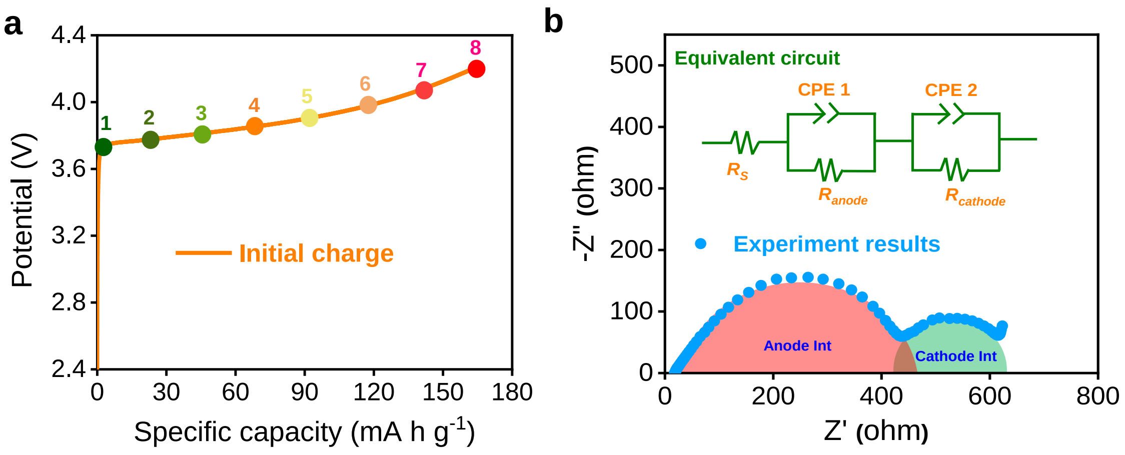

Supplementary Figure 5. In situ EIS of the NCM solid-state batteries during the initial charging process.

(a) Selected points with equal intervals in the charging process. (b) Equivalent circuit and illustration of the EIS model for the ASSLBs.

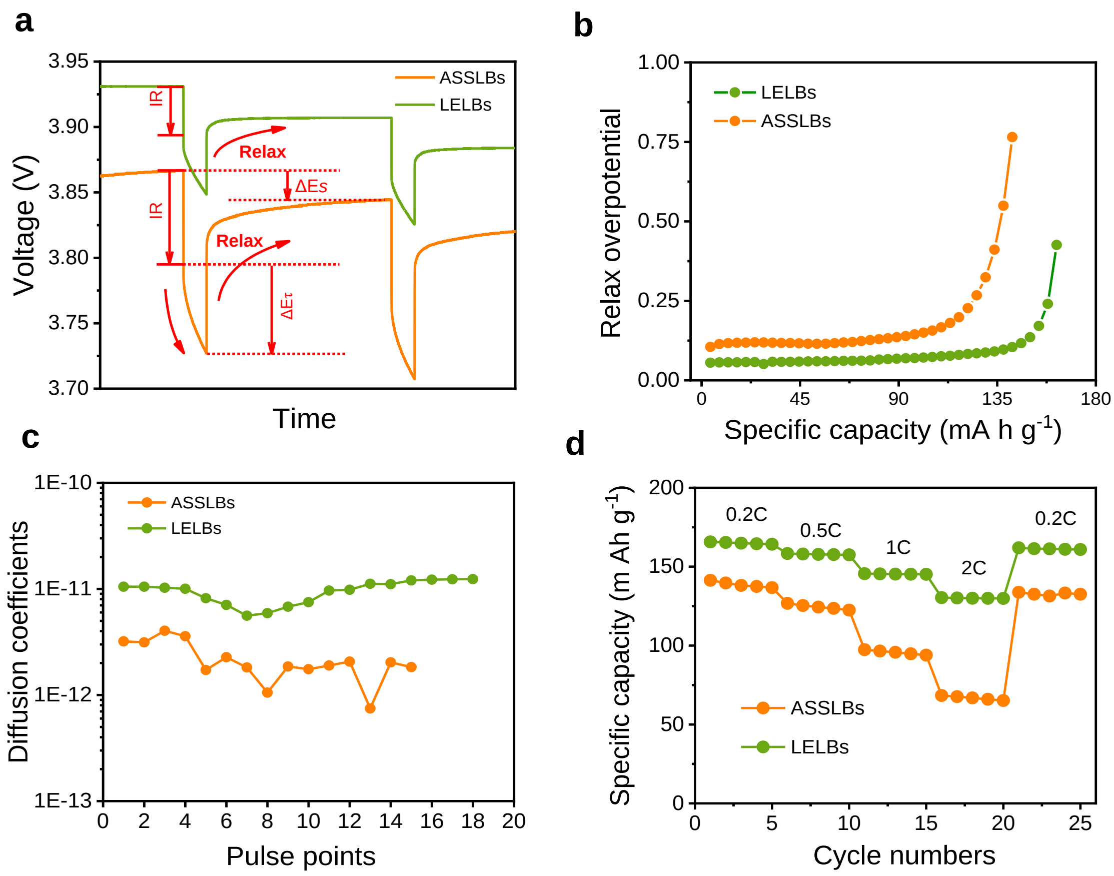

Supplementary Figure 6. Lithium diffusion evolution with the SOC changing and the effects on the rate permance. (a) Detailed voltage response during a single current pulse with time of LELBs and ASSLBs. (b) Comparison of relaxation overpotential at different points between the LELBs and the ASSLBs. (c) Calculated ion diffusion coefficients of LELBs and ASSLBs at different state of charge points. (d) The rate performance of the NCM electrodes in the LELBs and the ASSLBs ranging from 0.2 C to 2 C.

Supplementary Note 1

Calculation details for the GITT

The values of chemical diffusion coefficients (D_{Li^+}) in the cathode can be determined by applying the Fick's second law of diffusion. After a series of assumptions and simplifications, for sufficiently small current where overpotential for a single titration is small, the equation

for D_{Li+} can be written as:

$$D_{Li^+} = \frac{4}{\pi\tau}\left(\frac{m_B V_M}{M_B A}\right)^2 \left(\frac{\Delta E_S}{\Delta E_\tau}\right)^2 \qquad \text{Supplementary Equation (1)}$$

where m_B, V_M, M_B and A are the mass, the molar volume, the molecular weight, and the interface area of the electrode material. ΔEs and $\Delta E\tau$ is shown in the **Supplementary Figure 6a**. It may be noticed that $m_B V_M/M_B$ represents the volume of the electrode material. The molar volume (V_M) is assumed to remain stable as lithium content varying in NCM cathode or its possible change can be ignored. Based on the equation, the chemical diffusion coefficients of Li^+ in NCM electrodes show as a function of SOC is plotted in **Supplementary Figure 6c**.

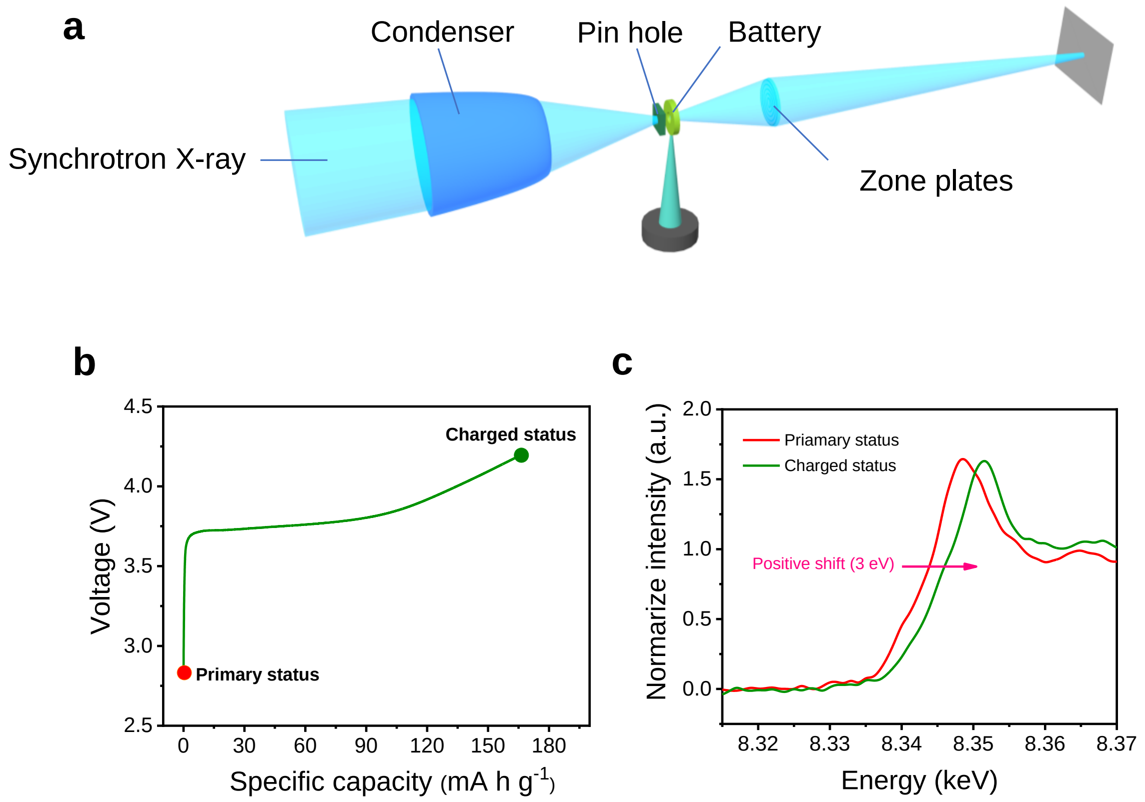

Supplementary Figure 7. (a) Schematic diagram of the measurement techniques of transmission synchrotron X-ray microscopic for solid-state batteries. Determination of quantitative standard of the XANES spectrum in ASSLBs. (b) Selected points of the pristine state and the final state. (c) The XANES spectrum reference obtained from the pristine state and the final state.

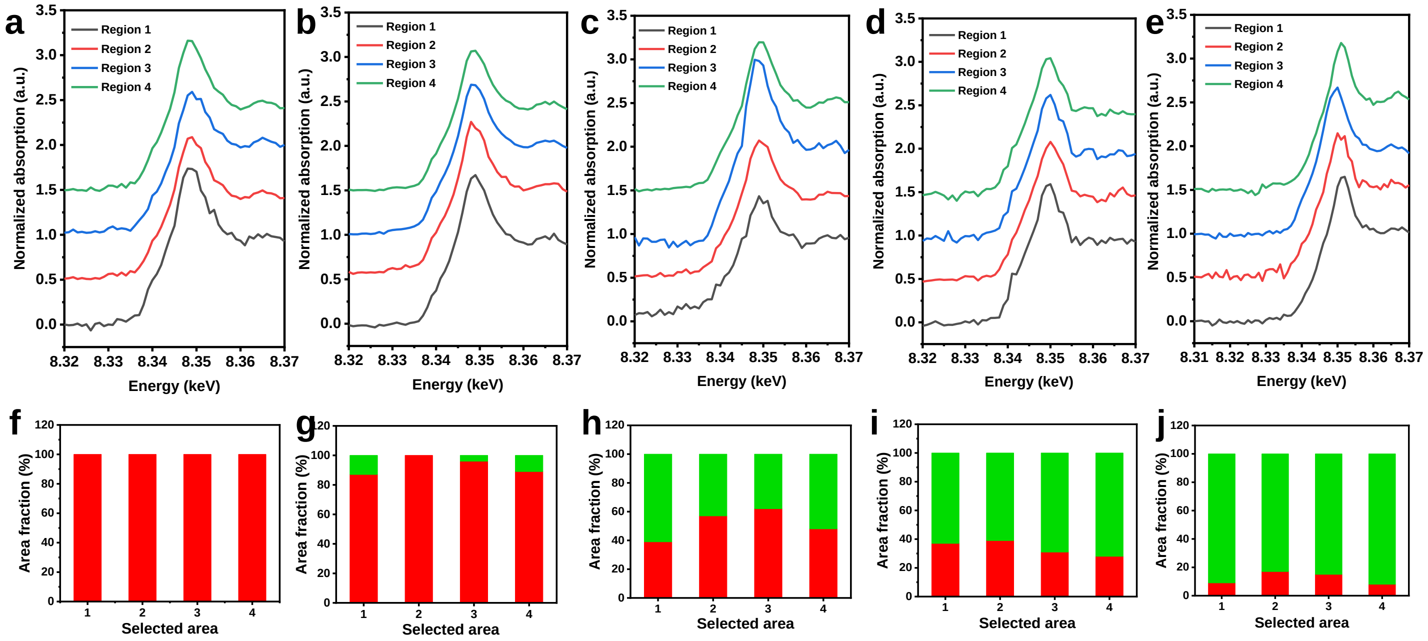

Supplementary Figure 8. Composition analysis of selected regions during the initial charging of ASSLBs. Extracted XANES results from region 1 to region 4, (a) pristine, (b) charging 30 min, (c) charging 60 min, (d) charging 90 min, (e) charging 120 min. Composition fractions in the particles, (f) pristine, (g) charging 30 min, (h) charging 60 min, (i) charging 90 min, (j) charging 120 min.

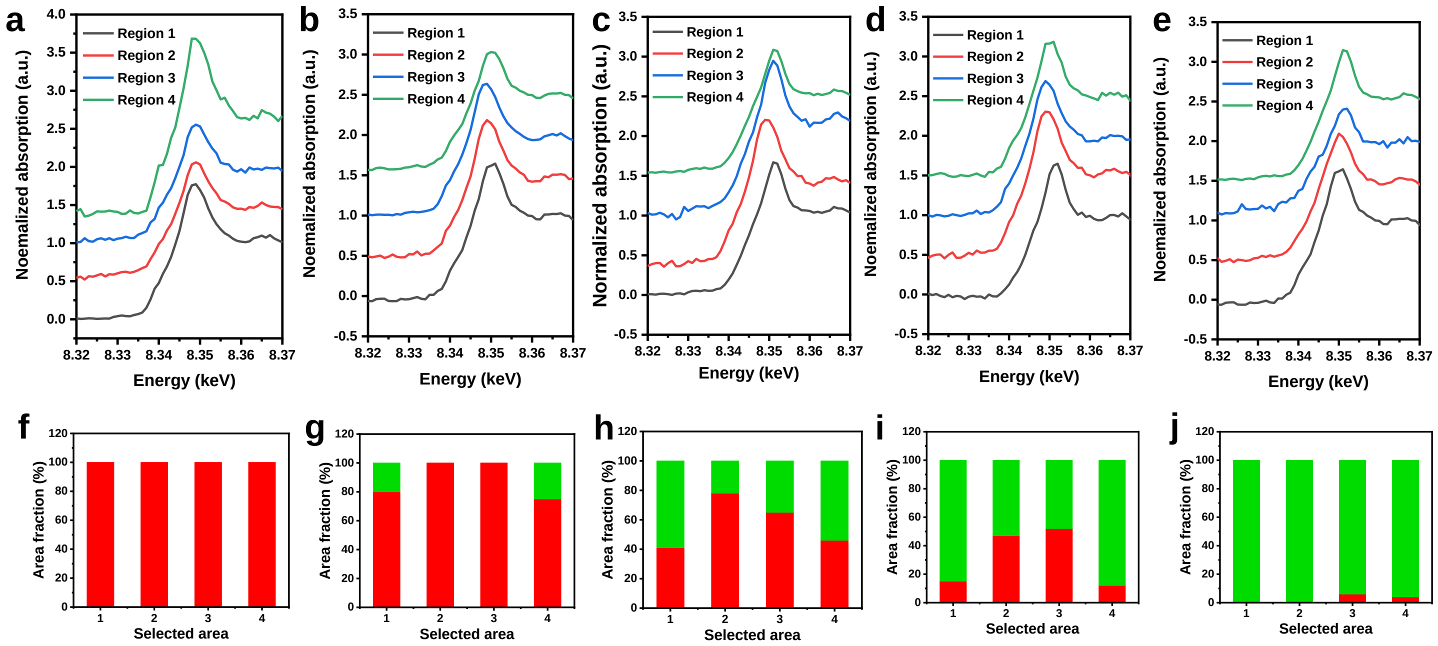

Supplementary Figure 9. Composition analysis of selected regions during the initial charging of LELBs. Extracted XANES results from region 1 to region 4, (a) pristine, (b) charging 30 min, (c) charging 60 min, (d) charging 90 min, (e) charging 120 min. Composition fractions in the particles, (f) pristine, (g) charging 30 min, (h) charging 60 min, (i) charging 90 min, (j) charging 120 min.

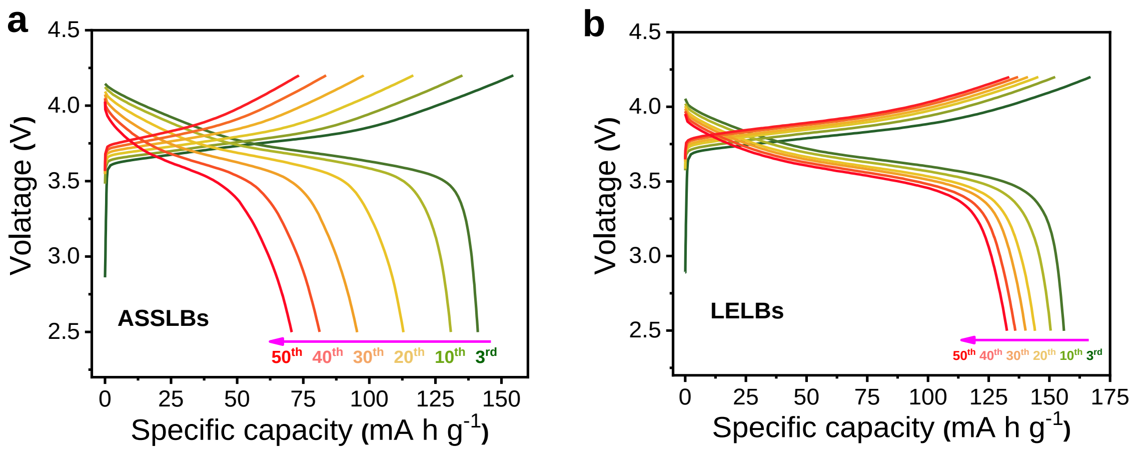

Supplementary Figure 10. Electrochemical performance of the ASSLBs and the LELBs during 50 cycles. The 3rd, 10th, 20th, 30th, 40th, and the 50th charging & discharging profiles of the (a) LELBs and (b) ASSLBs.

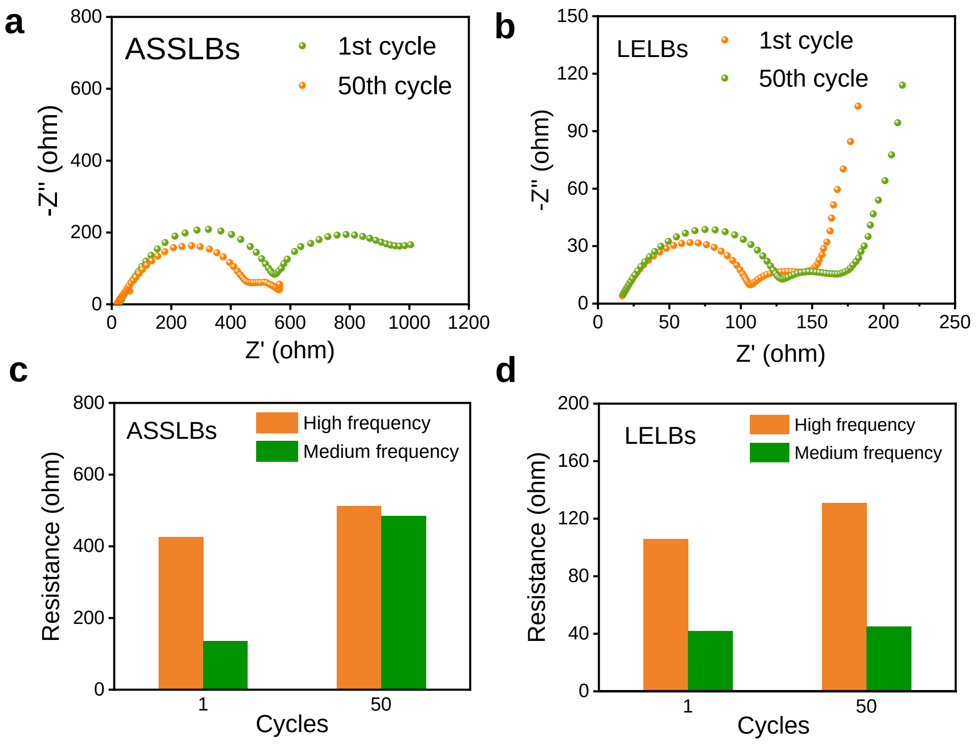

Supplementary Figure 11. Evolution of electrochemical resistance during 50 cycles. The EIS results in the 1st cycle and the 50th cycles of (a) ASSLBs and (b) LELBs. The corresponding fitting results of (c) ASSLBs and (d) LELBs.

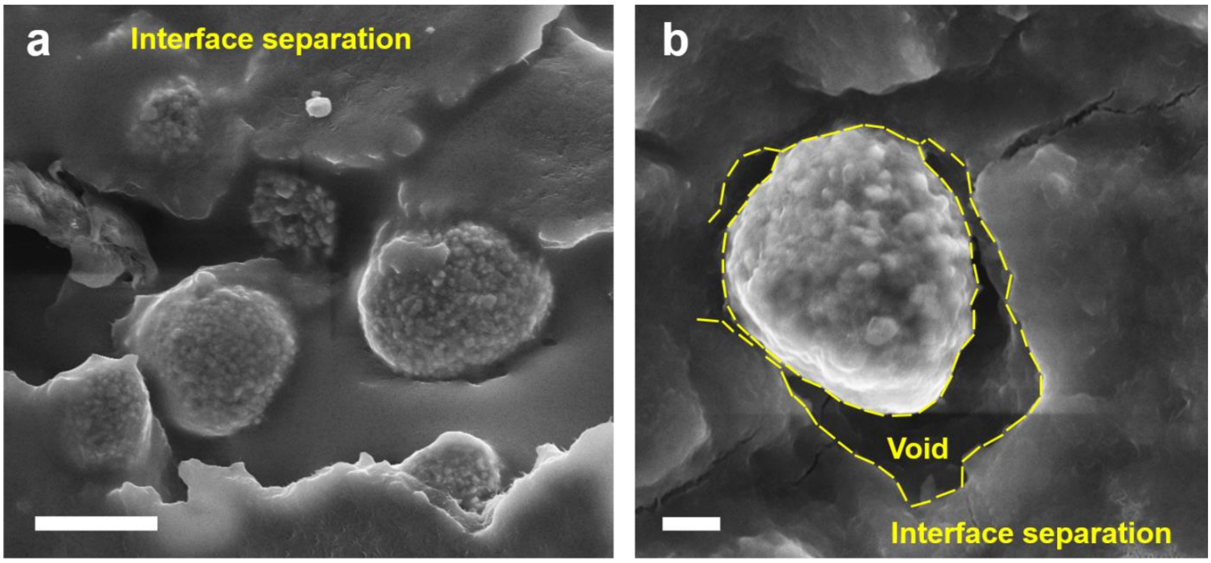

Supplementary Figure 12. Solid-solid interfaces voids after 50 cycles. SEM images of NCM electrode microstructure of ASSLBs with different magnifications after 50 cycles. (a) low magnifications. Scale bar, 10 μm. (b) High magnifications. Scale bar, 2 μm.

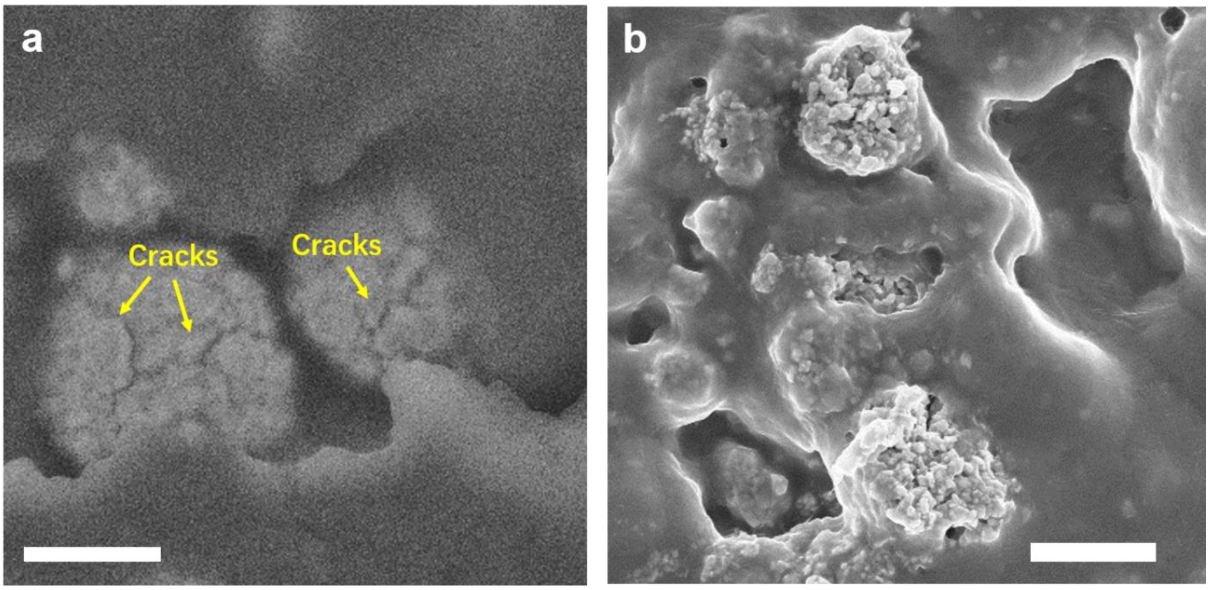

Supplementary Figure 13. Microcracks in the cathode particles of ASSLBs after 50 cycles. (a) SEM images of particles with obvious micro-cracks after 50 cycles. Scale bar, 10 µm. (b) SEM image of partial particles with obvious inner interfaces without covered by solid-state electrolytes after 50 cycles. Scale bar, 10 µm.

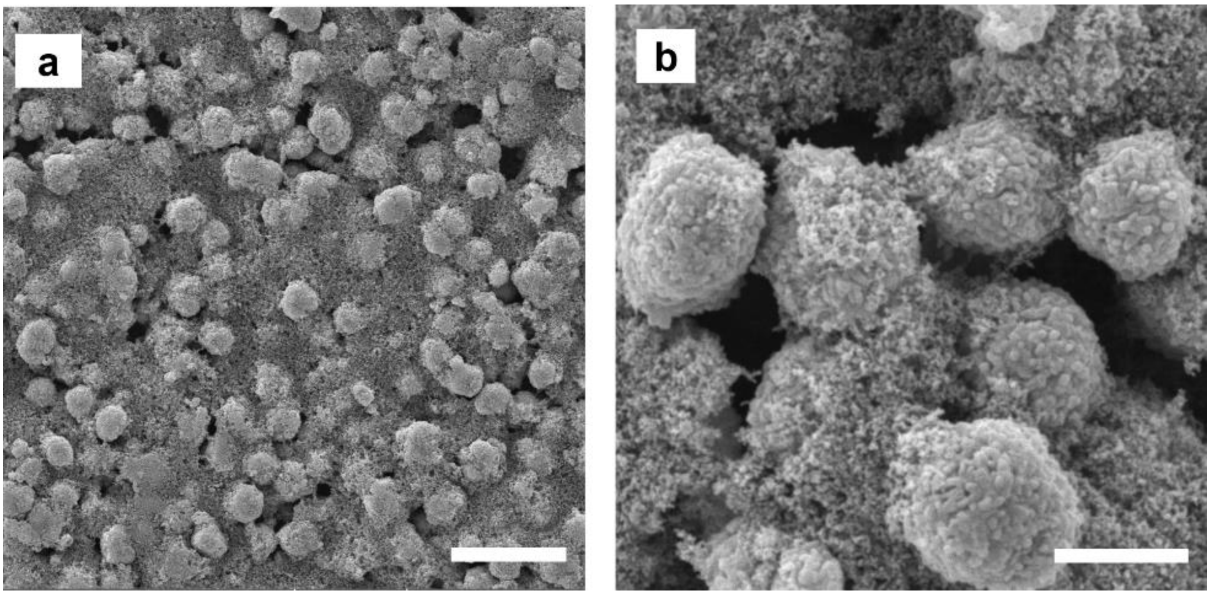

Supplementary Figure 14. SEM images of NCM cathodes cycled in LELBs after 50 cycles. (a) low-resolution image of the electrode. Scale bar, 30 µm. (b) High-resolution image of the electrode. Scale bar, 10 µm.

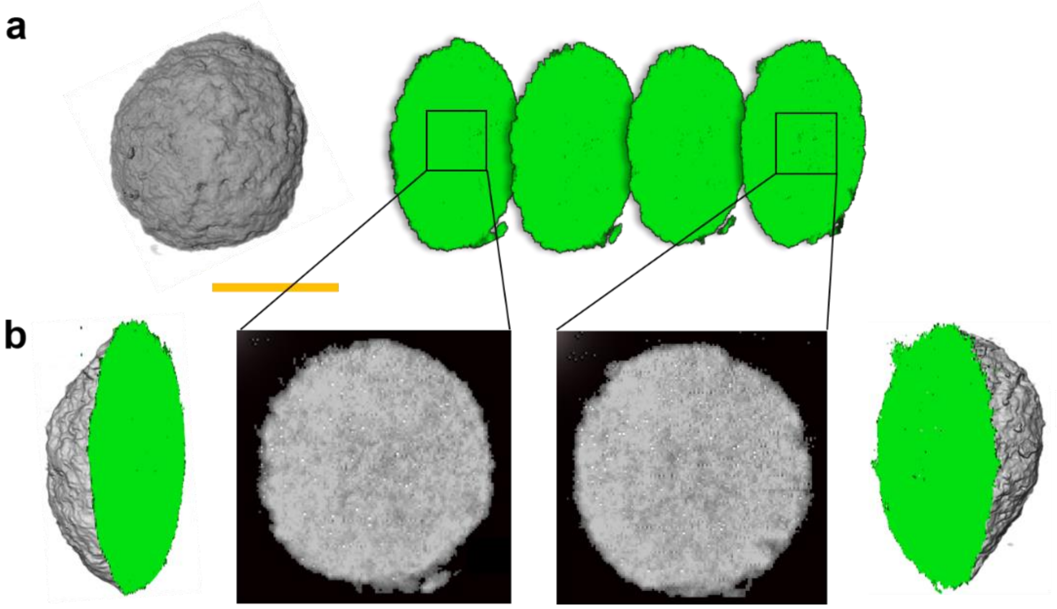

Supplementary Figure 15. Nanotomography of the cycled cathode particles in the LELBs. (a) 3D renderings and virtual slices of the secondary particle after 50 cycles. (b) Representative slices cut at different depths of the particle. Scale bar, 10 µm.

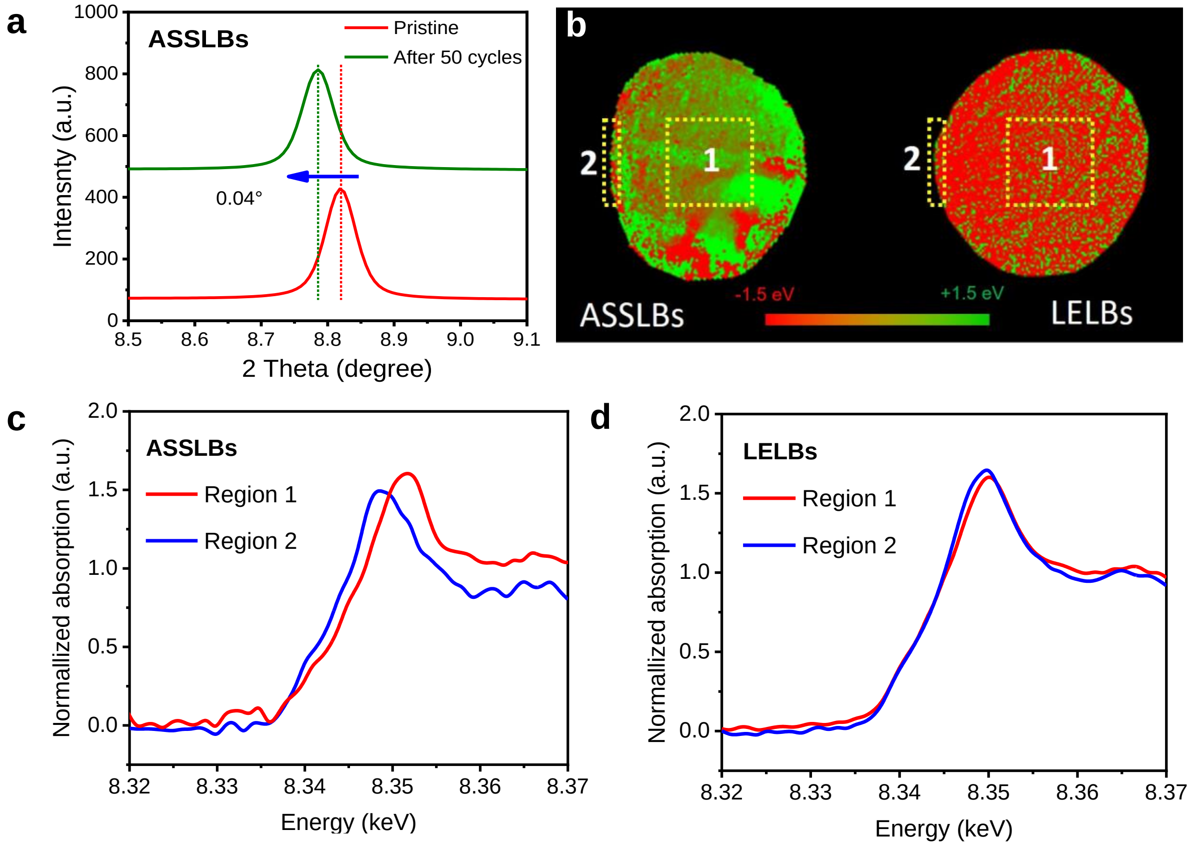

Supplementary Figure 16. Structure and chemistry evolution of the cycled NCM particles. (a) XRD patterns shift before and after 50 cycles in the ASSLBs. (b) 2D TXM-XANES mapping of cycled NCM particle in the ASSLBs and LELBs. (c) Average XANES spectra of the core region and interface region in the cracked particles within the ASSLBs. (d) Average XANES spectra of the core region and interface region in the particles within the LELBs.

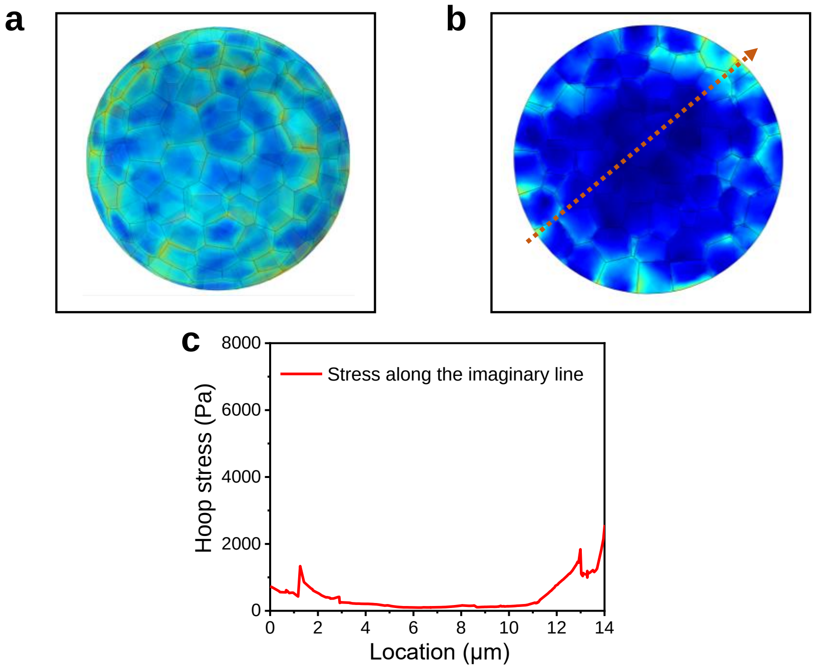

Supplementary Figure 17. Finite element modeling of the NCM secondary particles with half charging in the LELBs. The equivalent stress within the NCM particles. (a) 3D semitransparent view. (b) 2D view of the cross profile and the imaginary line. (c) Hoop stress within the NCM particles along the imaginary line.

Supplementary Note 2

Full numerical details used for the simulation process

The constitutive equation describing the stress-strain relation is given by

$$\varepsilon_{ij} = \varepsilon_{ij}^{e} + \varepsilon_{ij}^{c} = \frac{1}{E}\left[(1+v)\sigma_{ij} - v\sigma_{kk}\delta_{ij}\right] + \varepsilon_{ij}^{c} \qquad \text{Supplementary Equation (2)}$$

Where ε_{ij} is strain component, σ_{ij} is stress component, E is Young's modulus and v is Poisson's ratio, δ_{ij} is the Dirac delat function, ε_{ij}^{ch} is the chemical strain which is proportional to lithium concentration inside the particle,

$$\varepsilon_{ij}^{c} = (c - c_{ref})\beta_{ij} \qquad \text{Supplementary Equation (3)}$$

Where β_{ij} represents the lithiation expansion coefficients, *c* is the lithium concentration, and c_{ref} is a reference strain-free concentration. The fixed boundary conditions applied to the center and near the boundary of the current collector particles, free boundary conditions to the surface of the particles.

$$\sigma_r|_{r=R} = 0$$

$$u(r) = 0$$

Supplementary Equation (4)

According to the Fick second diffusion law, the diffusion equation is obtained as the governing equation for the Li diffusion, with the boundary conditions listed as below

$$\frac{\partial c}{\partial t} = D\nabla^2 c$$ Supplementary Equation (5)

$$c(r,0) = c_0$$ Supplementary Equation (6)

$$\nabla c(0,t) = 0$$ Supplementary Equation (7)

$$J(r_p,t) = \frac{i_{loc}}{F} = \frac{c_{max}c_i}{\mathrm{a}_v(3600s)}$$ Supplementary Equation (8)

Where *D* is the solid diffusion coefficient, c_{max} is the maximum lithium concentration, a_v is a specific surface area of the spherical particle, and c_i means the C-rate.

Here, the ion transport process was calculated by the transient solution, where the slver is PARDISO. The mechanical processes were handled by the steady-state solution with the MUMPS solver. All the calculations were operated in a high-performance compute with a 24-core processor and 128G ram for approximately 25 hours.

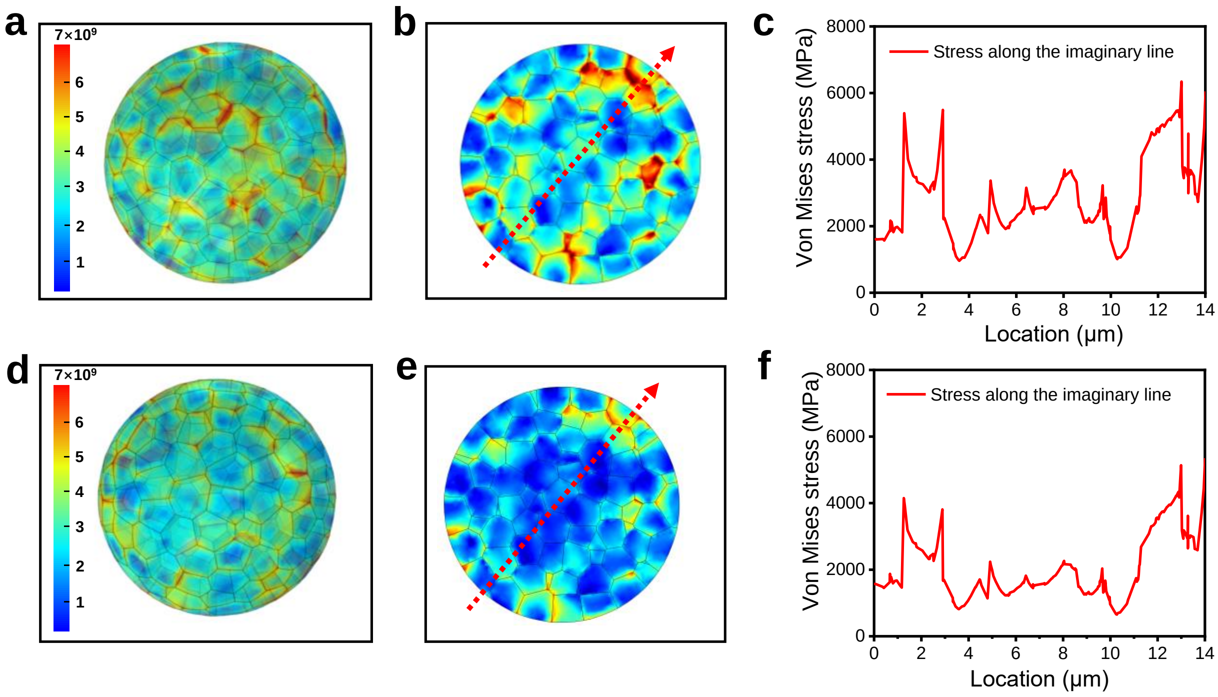

Supplementary Figure 18. Finite element modeling of the NCM secondary particles with complete charging. The equivalent stress within the NCM particles in the ASSLBs. (a) 3D semitransparent view. (b) 2D view of the cross profile and the imaginary line. (c) Hoop stress within the NCM particles along the imaginary line. The equivalent stress within the NCM particles in the LELBs. (d) 3D semitransparent view. (e) 2D view of the cross profile and the imaginary line. (f) Hoop stress within the NCM particles along the imaginary line.

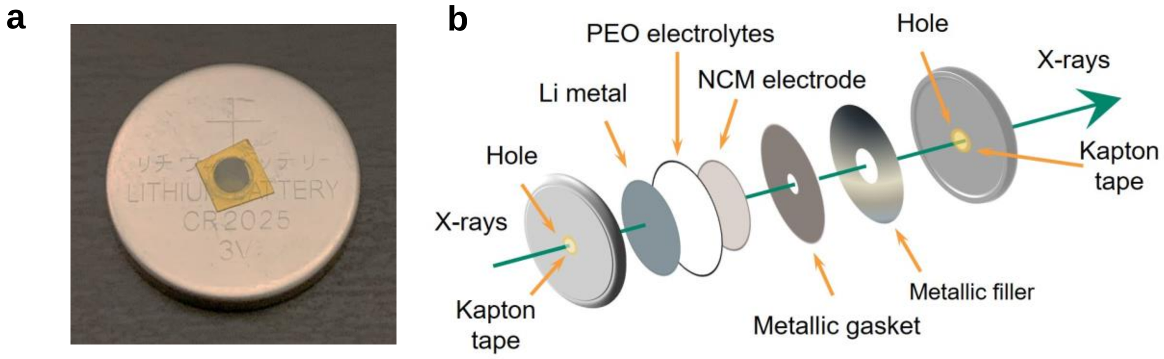

Supplementary Figure 19. The model cell and illustration for in operando TXM experiments. (a) The perforated coin cell employed for the operando studies. (b) Illustration of the operando cell containing the NCM electrode and all the other key components of a realistic battery.

Supplementary Table 1. Mechanical constants of NCM and LiNCM

	NCM	LiNCM
E_1	186.7 GPa	229.3 GP
E_2	186.7 GPa	229.3 GPa
E_3	54.2 GPa	152 GPa
V_1	0.3	0.3
V_2	0.24	0.24
V_3	0.24	0.24
G_{12}	71.5 GPa	95.3 GPa
G_{23}	20.8 GPa	63.2 GPa
G_{31}	20.8 GPa	63.2 GPa
